# Thyroidectomy Complications after Ligasure Small Jaw Use, Case Series, Prospective Study

**DOI:** 10.2174/0115748871336355250420013730

**Published:** 2025-05-07

**Authors:** Mohammad Bukhetan Alharbi

**Affiliations:** 1 General Surgery, Department of Surgery, Medical College, Imam Mohammad Ibn Saud Islamic University (IMSIU), Riyadh, Saudi Arabia

**Keywords:** Ligasure, thyroidectomy, parathyroid, recurrent laryngeal nerve, hypocalcaemia, injury

## Abstract

**Introduction:**

The Ligasure small jaw (LISJ) is believed to provide several benefits for thyroid surgery. The use of LISJ by non-expert surgeons or those who erroneously assume its extensive usage can reduce postoperative bleeding may, in reality, pose a considerable risk of additional severe consequences.

**Methods:**

Between March and December of 2022, I analyzed complications following total thyroidectomy (TTH) case series for 10 patients who underwent surgery for papillary thyroid cancer by one qualified surgeon using LISJ. Cohorts were evaluated prospectively including hypocalcemia (HC) and recurrent laryngeal nerve (RLND) palsy.

**Results:**

All 10 patients experienced postoperative hypocalcemia (HC), necessitating a postoperative calcium supplement with vitamin D. 9/10 (90%) patients had temporary HC, one patient (10%) had persistent HC. One patient (10%) suffered stridor after extubation, with RLND palsy.

**Discussion:**

Using the Ligasure small jaw in thyroid surgery necessitates greater awareness of the potential hazards associated with thermal injury, particularly the risk of compromising blood supply to the parathyroid gland and damaging the Recurrent Laryngeal Nerve (RLN).

**Conclusion:**

The Ligasure Small Jaw serves as an effective hemostatic device, but it requires more attention in complete thyroidectomy. During a complete thyroidectomy, the Ligasure Small Jaw's lateral heating action may affect delicate structures more often than reported. Such damage could lead to hypocalcemia, which could be temporary or permanent, and recurrent laryngeal nerve palsy. More research would provide greater insights into the anticipated complication rates, particularly when conducted by less experienced specialized surgeons.

## 1 INTRODUCTION

16.6% of patients had hypoparathyroidism after TTH. It appears to be straightforwardly relative to the magnitude of operation, underscoring the need to locate and protect the parathyroid glands throughout operation [1].

Soon following the surgical removal of the thyroid, subjective, nonspecific upper aerodigestive problems are prevalent. Injuries to the extrinsic perithyroidal neural plexus innervating the pharyngeal and laryngeal tissues could be responsible for these complaints [2].

The majority of otolaryngologists in the United States do not yet disclose routine use of RLND stimulation. Experience and practice of the operator   affected   intraoperative RLND monitoring far beyond operative workload [3].

Following complete thyroidectomy, HC is a significant problem. During TTH, avoiding postoperative HC necessitates precise operative methods, diagnosis, and conservation of the parathyroid glands' vasculature. The frequency of temporary HC was 7%, but the rate of persistent HC was 0.11% [4]. There was a 1.1% incidence of RLND damage. RLND Injury was not substantially linked with sociodemographic characteristics, etiology (benign *versus* malignant), or the amount of thyroidectomy [5].

The influence of gender, maturity, and histopathology on RLN palsy and iatrogenic HC was non-existent. The prevalence of RLN palsy and iatrogenic HC was considerably higher in patients with cancerous thyroid nodules. A comprehensive understanding of anatomy can lessen the occurrence of these issues [6].

Detection of the RLND using various procedures was comparable in thyroidectomies in which the RLND was identified at the area of the inferior thyroid artery or the level of Berry's ligament. Nevertheless, transient HC was much more prevalent in the initial group of thyroidectomy patients with RLND identification [7].

In addition, the incidence of late problems increases with age, even though a skilled surgeon can lower the probability of both initially and later consequences [8].

The unpredictability of surgical complications of thyroidectomies with blood clots poses a substantial hazard to patients and has major medical or legal consequences. Occurrences of post-surgical hemorrhage, unilateral vocal cord paralysis, lung infection, and surgical site infection [9].

The bipolar radiofrequency ablation equipment that is compatible with ordinary reusable bipolar cautery forceps is a simple, secure, and time-saving auxiliary for thyroid surgery that is just as successful as expensive devices such as the ultrasonic scalpel [10].

Furthermore, there was no important variation in operation period or surgical clinical outcomes between the bipolar energy sealing system and ultrasonic coagulation groups. The only important variation in expenses was between operators, regardless of the chosen equipment [11].

Safety of the harmonic and ligature has been evaluated. LISJ showed twice the complication rate of RLND palsy. But almost similar complications of HC about the harmonic scalpel [12]. Although no clinical trials showed comparison to the traditional technique of ties and micro clips for haemostasis with bipolar cautery.

Objective of the study: can LISJ be used safely without a clear guideline for its use in thyroid surgery.

## 2 METHODOLOGY

During March 2022 and December 2022, I evaluated postoperative complications after TTH. King Salman Hospital in Hail, Saudi Arabia, was evaluated prospectively, a case series of total thyroidectomy including HC, RLND injury.

10 patients underwent surgery for TTH for thyroid cancer, which was diagnosed preoperatively with fine needle aspiration and fit the criteria for total thyroidectomy. LISJ was used for those patients between November 2022 and December 2022. Given that our patients come from remote areas and plan to continue their medical care in tertiary centers outside our health system, it was challenging to maintain a longer period of follow-up. A single, qualified surgical oncologist surgeon performed all procedures. The limited patient volume with the same clinicopathological features in our small geographical area precluded the performance of a larger study volume.

The variables of interest were biochemical postoperative HC, persistent after discharge, and clinical RLND injury. Assessment of bias was not applicable due to a limited number of cases.

## 3 RESULTS

The follow-up of all those patients showed features of biochemical HC for total calcium (normal range 2.15 to 2.55 mmol/L) and ionized calcium (normal range 1.05-1.3 mmol/L), which was managed by Ca supplement. One patient has stridor and a low voice pitch after extubation.

All patients 10/10 patients (100%) suffered from postoperative HC with Ca supplement with Vitamin D, 9/10 (90%) patients had features of temporary (HC) patients missed follow-up after surgery, 4 patients had 2 different readings of HC, 2 patients had 4 readings of HC. One patient had 11 readings and the next had 19, suggestive of persistent (HC) 1/10 (10%). All Patients had papillary thyroid cancer, without retrosternal extension, or lymph node involvement, Table **1**.

During the same surgical admission, we evaluated the short-term RLND. Long-term injury assessment was not feasible because all patients live in remote areas with limited accessibility for patients to have proper long-term RLND injury evaluation, one patient showed RLND 1/10 (10%), as he had stridor during extubation.

The thyroidectomy was performed with the standard techniques of total thyroidectomy utilizing LISJ by a single surgeon; conventional techniques were not compared to LISJ due to the surgeon's preference for LISJ.

## 4 DISCUSSION

Female gender, neck dissection, parathyroidectomy, thyroid malignancy, multinodular goitres, and thyroiditis are identified as risk factors for postoperatively HC [13].

The patient's surgical complications can be reduced with anatomical awareness, excellent surgical skills, and an efficient strategy for the handling of complications [14]. If doctors are knowledgeable about the specifics of the surgical approach and limit injury to neighboring structures, surgery may be harmless [15].

81.4% of patients have four parathyroid glands, as indicated by the number of parathyroid glands. There are a total of 15.9% of misplaced parathyroid glands, with 11.6% in the neck and 4.9% in the mediastinum. 51.7% of misplaced parathyroid glands in the neck are localized in the retroesophageal/paraesophageal space or in the thyroid gland [16].

Left superior, 15.7%; left inferior, 31.3%; right superior, 15.8%; and right inferior, 37.2%. Considerably more inferior parathyroid glands were anomalous (68.5%) than their superior counterparts (31.5%). 43.4% of solitary anomalous parathyroid glands in males were located in the right inferior location. There was no difference of statistical significance in laterality [17].

After thyroidectomy, HC was widespread postoperatively, although hypomagnesaemia was uncommon. After operation, hypomagnesaemia and a decrease in magnesium levels were related to HC, but not clinical HC. Measuring magnesium levels is important in cases of severe or prolonged HC [18].

For sutureless thyroidectomy, the LISJ approach with double or overlapped sealing is helpful. During LISJ activation, however, surgeons must be mindful of tissue contraction that may decrease the space between the LISJ and the RLND and raise the risk of damage [19]. Experienced surgeons are crucial for reducing the rate of complications [20]. Compared to the conventional technique, variations in total and ionized calcium levels in patients with LISJ thyroidectomy are minimal. Using LISJ looks to significantly reduce surgical duration, bleeding during surgery, and blood in the drain after surgery compared to the traditional technique [21, 22]. In some reports LISJ is more advantageous than the conventional approach [23].

When LISJ was applied with tight proximity, RLND roots were subjected to higher degrees. Due to their effects on both EMG and temperature, the findings show that all these LISJ should not be put within 1 to 3 mm of the RLND [24].

Based on a functional assessment of electromyography in real time, the secure spacing for ligature small mandible stimulation was determined to be 2 mm. Much farther touch between the ligature small jaw and the RLND was harmless if the ligature small jaw was cooled for at least 2 seconds or by a contact muscle technique. Using the ligature small mandible with these time and location constraints in consideration will prevent RLND damage [25].

The right RLND branched off of the vagus nerve at or below the level of T1-T2. After winding around the subclavian artery, the right RLND becomes embedded in the tracheoesophageal fascia more than 0.5 cm below C7-T1. Before traveling between the trachea and the thyroid, the RLND travels superiorly, slightly anterior to the tracheoesophageal groove. In 82 percent of right-sided dissections, the RLND entered the larynx at C6-C7 or below. After circling the aortic arch, the left RLND is embedded in the tracheoesophageal fascia below the T2 level. Before traveling between the trachea and thyroid, the nerve travels slightly anterior to the tracheoesophageal fissure and along the tracheoesophageal fascia. In all dissections of the left side, the RLND entered the larynx at or below C6-C7 [26].

The frequency of the RLND interlacing with the inferior thyroid artery was 22.3% because of inherent variability. 4 RLND and the inferior thyroid artery have a highly variable association. Individual variation is typically categorized into six groups [27]. Which still makes the question of LISJ safety under concern, especially for junior surgeons.

Sixty-three percent of the RLNDss were deep to the inferior thyroid artery, thirty percent were superficial to the artery, and seven percent were located between the arterial segments. A nerve branching was discovered in sixty percent of patients. In forty percent, no nerve branching was detected. Throughout surgeries, 1% of patients were proven to have non-RLNDs [28].

## CONCLUSION

Although the Ligasure Small Jaw serves as an effective hemostatic device in the surgical domain, complete thyroidectomy necessitates further caution. The Ligasure Small Jaw's lateral heating action during a complete thyroidectomy may affect delicate structures. Hypocalcemia, which can be temporary or permanent, and recurrent laryngeal nerve palsy may happen more often than what has been written about. More randomized clinical trials would provide greater insights into the anticipated complication rates, particularly when conducted by general surgeons or less experienced specialized surgeons.

## STUDY LIMITATIONS

 The study is limited because it is a one-center study with a small number of patients, and progress is not possible without clearer guidelines. However, it raises a significant concern about the expansion of new surgical devices without clear guidelines or transparent results. 

## AUTHORS’ CONTRIBUTIONS

The author confirms sole responsibility for the following: study conception and design, data collection, analysis and interpretation of results, and manuscript preparation.

## Figures and Tables

**Table 1:** Post total thyroidectomy complication, due to thyroid cancer, using ligasure.

**Number**	**Procedure**	**Complication 1**	**Complication 2**
1	Total Thyroidectomy	Strider, low voice pitch	Hypocalcemia
2	Total Thyroidectomy	-	Hypocalcemia 2 reading, still on Ca supplement
3	Total Thyroidectomy	-	Hypocalcemia
4	Total Thyroidectomy	-	Hypocalcemia 2 reading, still on Ca supplement
5	Total Thyroidectomy	-	Hypocalcemia 4 reading, still on Ca supplement/IV Ca
6	Total Thyroidectomy	-	Hypocalcemia 11 reading, still on Ca supplement/IV Ca
7	Total Thyroidectomy	-	Hypocalcemia 2 reading, still on Ca supplement
8	Total Thyroidectomy	-	Hypocalcemia
9	Total Thyroidectomy	-	Hypocalcemia 4 reading, still on Ca supplement/IV Ca
10	Total Thyroidectomy	-	Hypocalcemia 19 reading, still on Ca supplement/IV Ca

## Data Availability

The data and supportive information are available within the article.

## LIST OF ABBREVIATIONS

HC: Hypocalcemia
LISJ: Ligasure Small Jaw
RLND: Recurrent Laryngeal Nerve
TTH: Total Thyroidectomy

## STANDARDS OF REPORTING

STROBE guidelines were followed.
